# Correlations of pilot trainees' brainwave dynamics with subjective performance evaluations: insights from EEG microstate analysis

**DOI:** 10.3389/fnrgo.2025.1472693

**Published:** 2025-03-05

**Authors:** Mengting Zhao, Andrew Law, Chang Su, Sion Jennings, Alain Bourgon, Wenjun Jia, Marie-Hélène Larose, David Bowness, Yong Zeng

**Affiliations:** ^1^Concordia Institute for Information Systems Engineering, Gina Cody School of Engineering and Computer Science, Concordia University, Montreal, QC, Canada; ^2^Flight Research Laboratory, Aerospace Research Centre, National Research Council of Canada, Ottawa, ON, Canada; ^3^CAE Inc., St-Laurent, QC, Canada; ^4^Marinvent Corporation, St-Bruno, QC, Canada

**Keywords:** aircraft control evaluations, brain dynamics, EEG microstate analysis, correlation analysis, cognitive functions, pilot training, learning processes

## Abstract

**Objective:**

This study aims to investigate the relationship between the subjective performance evaluations on pilot trainees' aircraft control abilities and their brainwave dynamics reflected in the results from EEG microstate analysis. Specifically, we seek to identify correlations between distinct microstate patterns and each dimension included in the subjective flight control evaluations, shedding light on the neurophysiological mechanisms underlying aviation expertise and possible directions for future improvements in pilot training.

**Background:**

Proficiency in aircraft control is crucial for aviation safety and modern aviation where pilots need to maneuver aircraft through an array of situations, ranging from routine takeoffs and landings to complex weather conditions and emergencies. However, the neurophysiological aspects of aviation expertise remain largely unexplored. This research bridges the gap by examining the relationship between pilot trainees' specific brainwave patterns and their subjective evaluations of flight control levels, offering insights into the cognitive underpinnings of pilot skill efficiency and development.

**Method:**

EEG microstate analysis was employed to examine the brainwave dynamics of pilot trainees while they performed aircraft control tasks under a flight simulator-based pilot training process. Trainees' control performance was evaluated by experienced instructors across five dimensions and their EEG data were analyzed to investigate the associations between the parameters of specific microstates with successful aircraft control.

**Results:**

The experimental results revealed significant associations between aircraft control levels and the parameters of distinct EEG microstates. Notably, these associations varied across control dimensions, highlighting the multifaceted nature of control proficiency. Noteworthy correlations included positive correlations between microstate class E and class G with aircraft control, emphasizing the role of attentional processes, perceptual integration, working memory, cognitive flexibility, decision-making, and executive control in aviation expertise. Conversely, negative correlations between microstate class C and class F with aircraft control indicated links between pilot trainees' cognitive control and their control performance on flight tasks.

**Conclusion:**

The findings underscore the multidimensional nature of aircraft control proficiency and emphasize the significance of attentional and cognitive processes in achieving aviation expertise. These neurophysiological markers offer a basis for designing targeted pilot training programs and interventions to enhance trainees' aircraft control skills.

## 1 Introduction

The aviation domain demands unparalleled levels of cognitive processing, motor coordination, and conscious attention to ensure the safe and efficient execution of flight operations (Sikora et al., 2020; Wickens et al., 2002; Lintern et al., 1990; Dehais et al., 2017; Prokopczyk and Wochyński, 2022). Modern aviation demands pilots to maneuver aircraft through an array of situations, ranging from routine takeoffs and landings to complex weather conditions and emergencies. The mastery of aircraft control skills emerges as an unequivocally vital aspect of aviation expertise, directly influencing the pilot's capacity to navigate and respond proficiently to dynamic and challenging flight conditions (Zaal and Mobertz, 2017; Feary, 2018; Prokopczyk and Wochyński, 2022). Skilled control over an aircraft enables pilots to maintain precise trajectories, stabilize flight during turbulent weather, and conduct critical maneuvers with confidence and precision. It is through proficient aircraft control and decision-making skills that pilots are equipped to avert potential dangers, respond effectively to unexpected situations, and ensure the safety of passengers and crew members (Taylor et al., 2005; Strickland et al., 2019; Gordon et al., 2016). Effective control skills, developed through rigorous training and experience, empower pilots to maintain precise navigation, efficient fuel consumption, and adherence to designated flight paths, contributing to efficient air traffic management and reduced operational costs. Research findings have associated the level of expertise with better performance in flight control (Taylor et al., 2007; Kennedy et al., 2010). The proficiency of pilots' aircraft control skills under diverse flight scenarios constitutes pivotal determinants of aviation safety and operational success.

Some applications have successfully quantified pilots' or vehicle drivers' cognitive changes from their physiological signals (Masi et al., 2023). Among these signals, electroencephalogram (EEG) has been the focus of extensive research interest (Sibi et al., 2017; Causse et al., 2019; Borghini et al., 2017; Balters et al., 2021; Zhao et al., 2020). Given its high temporal resolution, time-based and frequency-based EEG features have been applied to detect mental and functional abnormalities, as well as cognitive and affective states under external or internal stimuli (Acharya et al., 2013; Pidgeon et al., 2016; Su et al., 2024). Moreover, EEG is considered a valuable tool for investigating temporal changes in trainees' brains without imposing additional workload or interference. For instance, a decline in alpha power has been witnessed in correlation with escalating cognitive workload (Stipacek et al., 2003; Gevins and Smith, 2003; Kamzanova et al., 2014). Substantial reductions in alpha activity were pinpointed in the fronto-central and parietal regions (Slobounov et al., 2000; Fairclough et al., 2005). On a parallel note, theta oscillations are speculated to contribute to working memory enhancement (Roux and Uhlhaas, 2014; Raghavachari et al., 2001; Tesche and Karhu, 2000; Jensen and Lisman, 1998), facilitation of cognitive control (Cavanagh and Frank, 2014), and orchestrating rhythmic shifts in spatial attention (Fiebelkorn and Kastner, 2019; Herweg et al., 2020). Empirical findings suggest a positive correlation between higher EEG theta power and efficacious information encoding and memory retrieval during memory tasks (Staudigl and Hanslmayr, 2013; Guderian and Düzel, 2005; Addante et al., 2011). However, the underlying neurophysiological mechanisms associated with skilled aviation control or aircraft control skills remain largely unexplored. Investigating the brain mechanisms associated with skilled aircraft control would shed light on the neural plasticity and adaptability underlying pilot training, offering insights into optimizing instructional methods and designing targeted interventions for enhanced pilot performance and safety (Zhao et al., 2024). EEG microstate analysis, as a method that allows for simultaneous investigation into the spatial properties and temporal dynamics of the human brain, was applied in this research as it appears to be an attractive method to be applied to aviation research. Unlike traditional EEG features, microstates capture scalp potential topography patterns and their changes over time (Lehmann et al., 1987; Pascual-Marqui et al., 1995).

Therefore, this research aims to bridge the gap between subjective evaluations of aircraft control expertise and neurophysiological understanding by applying EEG microstate analysis to explore the correlations between pilot trainees' control evaluations and brainwave dynamics. We conducted EEG microstate analysis to the pre-processed EEG data originally collected from a group of pilot trainees during a simulator-based pilot training process. Afterward, paired comparisons were conducted on both the EEG microstate features extracted from each computed microstate class across different training stages and the control performance evaluated by training instructors. Finally, we computed the Spearman correlation coefficients between the EEG microstate parameters and the five dimensions of control expertise covered in subjective evaluations.

## 2 Materials and methods

### 2.1 Experiment design and participants

**Figure 1:**
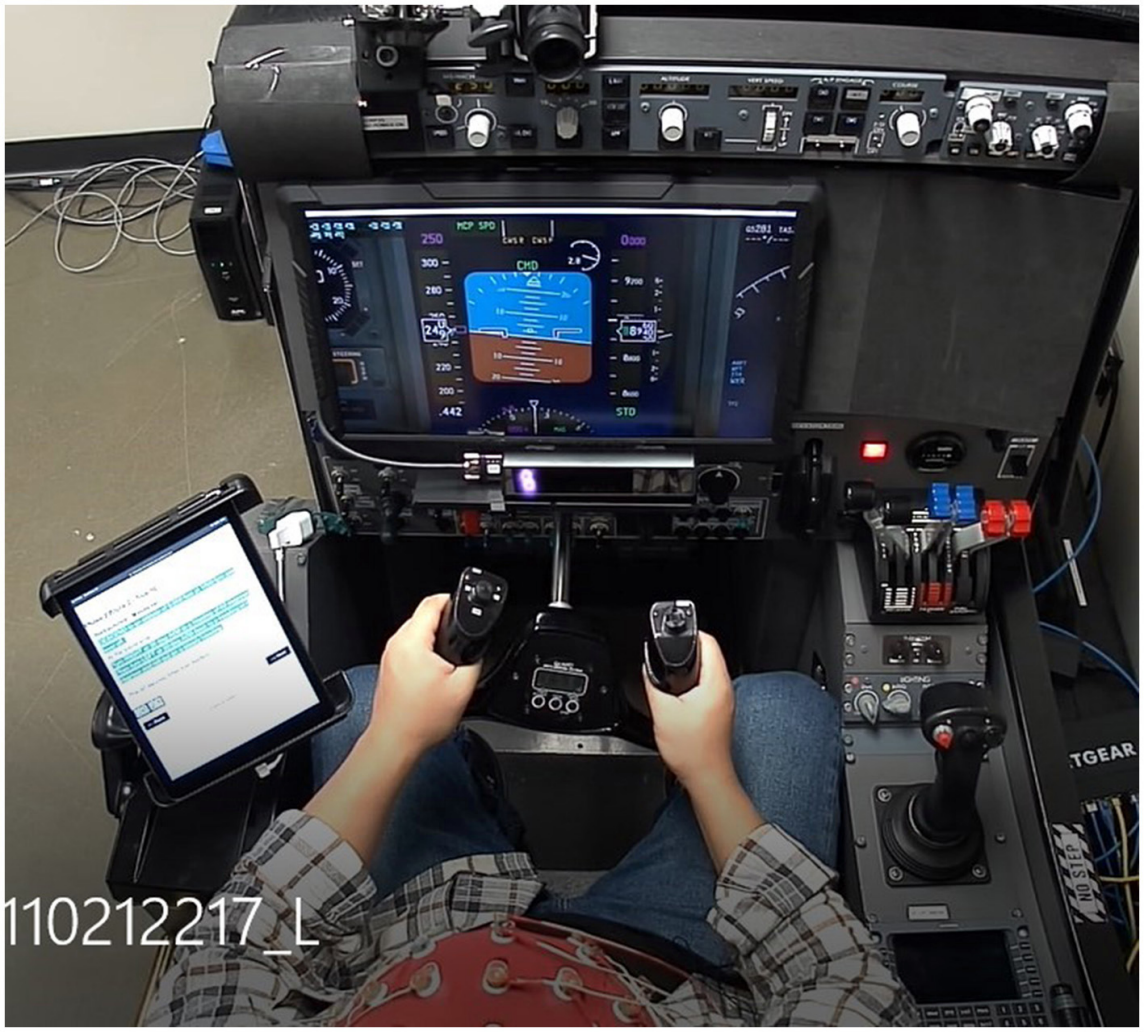
Flight simulator and the experimental settings viewed from the top camera.

**Figure 2:**
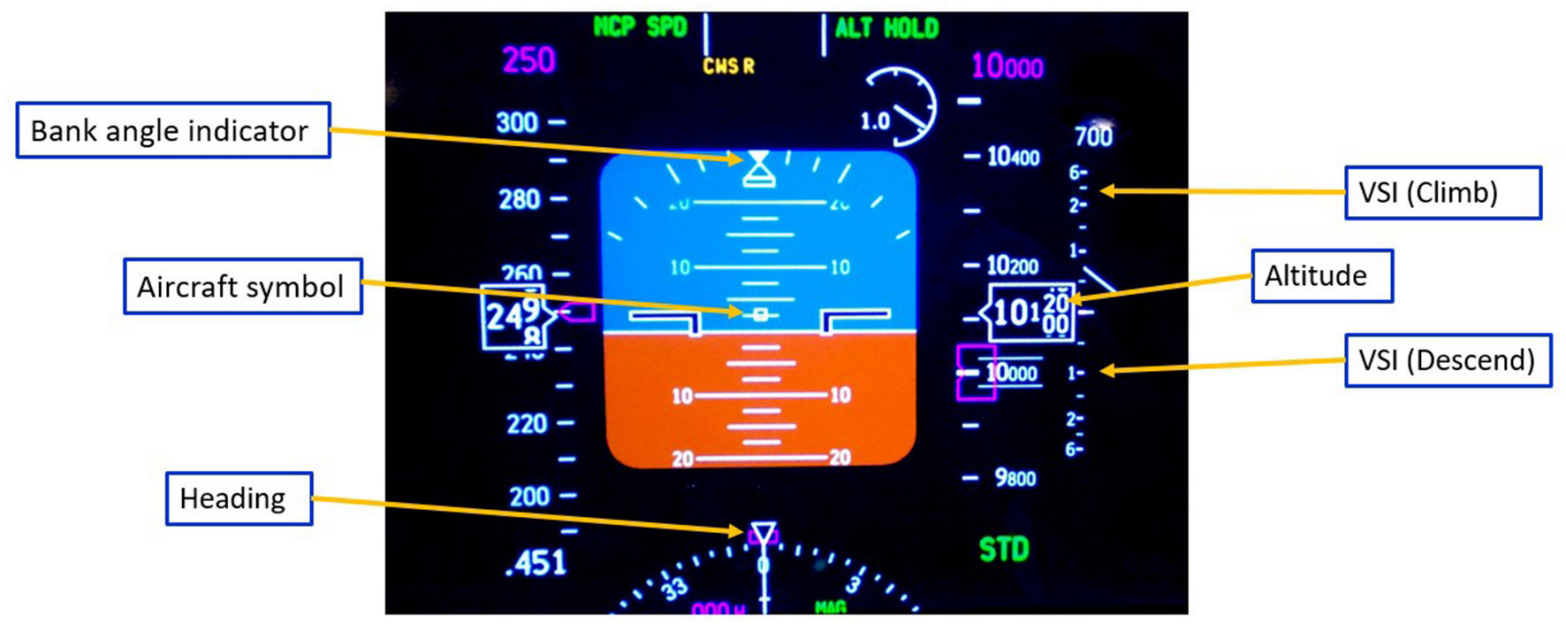
Information displayed on the primary flight display (PFD).

The experiment was conducted on a custom aircraft flight simulator as shown in Figure 1, which was built by Marinvent Corporation. The simulator reflected the dynamics of a Boeing 737, modeled using XPlane 11 from Laminar Research on a computer running the Windows 10 operating system. The simulator controls included a yoke, pedals, rudder, and throttle quadrant. During the experiment, participants did not have an out-the-window view but relied solely on a primary flight display (PFD) (Figure 2) for information about the aircraft's attitude, altitude, heading, climb rate, and speed. Participants used the yoke to control pitch and roll, while an autopilot managed the throttles (for speed maintenance) and pedals (for turn coordination), allowing participants to focus exclusively on operating the yoke.

The study recruited twenty-four participants (11 males, 13 females) aged 21 to 41, all in good health with no neurological or psychiatric disorders. Participants had normal vision or corrected-to-normal vision using contact lenses, as glasses were not permitted. Each participant received *$*100 CAD compensation, as well as *$*80 CAD for transportation expenses upon completion of experiments. Before commencing the pilot training process, all participants underwent several preparation steps to ensure a basic understanding of flight instruments and maneuvers. These steps included reading a flight briefing presentation, watching four training videos, and participating in a familiarization session in the simulator with the test director. In the simulator session, participants practiced the basic maneuvers explained in the videos and received instruction on the yoke as flight control and its effects on pitch and roll. Furthermore, participants were taught how to interpret control and performance indications on the Primary Flight Display (PFD) as illustrated in Figure 2, along with some fundamental flight control strategies. Informed consent forms were signed, and participants completed a questionnaire pertaining to the training tasks. Based on their responses, the experimenters conducted additional interviews with the participants to gather further information. More details of participants' demographic information could be found in Supplementary Table S1. Participants were asked to complete two separate resting sessions prior to the training process: one 2-min session with their eyes open, and another 2-min session with their eyes closed. The resting state sessions were combined with task sessions (training and practice) to compute global EEG microstate maps and were also used to baseline data for other physiological measurements, such as heart rate and GSR, which were reported in Darvishi-Bayazi et al. (2023) and Ruiz-Segura et al. (2024).

The pilot training process consisted of 22 sessions. The required maneuvers were provided to the participants through an iPad positioned in front of them, and the test director supervised the process. Each session consisted of a sequence of tasks. Firstly, participants were instructed to perform a Baseline task for a duration of 30 seconds. Subsequently, they were required to engage in a Trial task for a duration of 90 seconds. After completing each session, participants were prompted to assess their perceived workload by completing a NASA-TLX questionnaire on the iPad. During the Baseline tasks, participants were instructed to maintain straight and level flight at a constant heading and altitude. The maneuvers requested in these tasks remained the same for all participants. Following the completion of the Baseline task, the simulator was paused and reset, and participants were provided with instructions for the subsequent Trial stage. In the Trial task, participants were required to perform maneuvers of varying difficulty levels, which were categorized into three distinct levels: (1) a climb, descent, or turn in one direction; (2) a climb, descent, or turn with a reversal; and (3) a climb or descent with reversal combined with a turn in one direction; or a turn with reversal combined with a climb or descent.

The experimental protocol was approved by both the Concordia Human Research Ethics Committee and the research ethics board of the National Research Council Canada. All participants gave their written informed consent before the experiment and were financially compensated for the experiment regardless of their performance.

### 2.2 Experimental data collection

Once the pilot training process started, participants' physiological data and learning behaviors were recorded untill the end of the experiment. The recordings were controlled through the National Research Council's Integrated Physiological Monitoring System (IPMS) (Law et al., 2017) and were synchronized with the aircraft simulator using a network time protocol server. An iPad located to the left of the participant presented task instructions and collected NASA-TLX ratings and other questionnaire data (e.g., fatigue ratings). Instructions were presented and data were collected using Qualtrics software. Electroencephalogram (EEG) data were acquired using a 64-channel BioSemi ActiveTwo system, positioned following the international 10—20 system, and sampled at a rate of 2,048 Hz. Additionally, we also recorded other physiological data like ECG and GSR, while participants' learning behaviors were recorded using the training devices, along with three cameras placed at different angles (top, front, and side) to capture their actions. For comparative analysis, subjective real-time evaluation by an instructor was impossible due to COVID-19 health restrictions. Still, it was conducted post-experiment by a review of parametric data and video of the sessions.

The recorded experiment process consisted of 22 sessions, which were categorized into three stages to track different phases of the training process: Training (comprising 7 Baseline and 7 Trial tasks), PracticeA (comprising 8 Baseline and 8 Trial tasks), and PracticeB (comprising 7 Baseline and 7 Trial tasks). In the Training stage, participants received performance feedback from the test director and performed a standardized sequence of maneuvers. However, in Practice A and Practice B stages, participants did not receive feedback from the test director, and the sequence of maneuvers was pseudo-randomized among participants. The maneuvers were presented in sets of three, each set comprising one twizzle from Level 1, Level 2, and Level 3 difficulty levels. Additionally, the first trial task in a new set could not have the same difficulty level as the last trial task of the previous set. EEG signals were also collected during the resting sessions, which were later included in the computation of global microstate classes.

### 2.3 Data pre-processing

The collected EEG data underwent a series of preprocessing steps using the EEGLAB toolbox (Delorme and Makeig, 2004) in Matlab. The data were referenced to mastoids and then filtered using a zero-phase Hamming windowed-sinc FIR filter, with a frequency range of 1 to 40Hz, as EEG microstates are usually based on this frequency band (Jensen et al., 2007; Herrmann et al., 2004; Tallon-Baudry, 2009). Channels that met one or more of the following criteria below were identified as bad channels: (1) the channels that remained flat for more than 5 seconds; (2) channels with a correlation coefficient smaller than 0.8 with their neighboring channels; and (3) channels with amplitudes greater than 3 standard deviations from the mean. For artifact removal, we applied an automatic artifact removal process via MARA, the multiple artifact rejection algorithm (Winkler et al., 2011), to provide a consistent, objective approach to artifact rejection across all participants and datasets, reducing the variability that can arise from manual operations. MARA was applied to identify and remove the IC components (Makeig et al., 1995) having more than 40% chance to be labeled as artifacts (eye-blink, eye-movement, muscle-generated, and other artifacts). Moreover, the signals were segmented in 2-second epochs for detecting bad segments and bad local channels within each segment (Gabard-Durnam et al., 2018).

Bad local channels in each segment were detected using FASTER (Nolan et al., 2010) criteria (variance, median gradient, amplitude range, and deviation from mean amplitude) when one or more Z scores of four criteria were greater than 3 standard deviations from the mean, which were interpolated thereafter using spherical splines. Afterward, bad segments were identified and rejected when one or more criteria were satisfied: a channel' amplitude was higher than ±100μV; the single electrode probability across segments or the electrode group probability within segments was greater than 3 standard deviations from the mean. Finally, the isolated bad global channels were interpolated using spherical splines. The cleaned EEG signals were re-referenced to the average reference and were downsampled to 250 Hz.

### 2.4 EEG microstate analysis

Each microstate reflects an activation pattern in large-scale brain networks and represents a quasi-stable state of the brain (Lehmann et al., 1987). As reviewed in Michel and Koenig (2018), the four microstates (A, B, C, D) identified in most previous studies exhibited high similarity, and further research has focused on the associated functional role. For instance, microstate class C has been linked to the engagement of cognitive control networks, including the prefrontal cortex and anterior cingulate cortex (Khanna et al., 2015; Milz et al., 2016). Microstate class D has been associated with the dorsal attention network (Britz et al., 2010; Seitzman et al., 2017), while microstate class A and class B are associated with phonological process and visual processing supported by fMRI and EEG evidence (Seitzman et al., 2017; Milz et al., 2016). In the meantime, researchers have applied six or seven microstate classes to investigate different cognitive states including mental workload and cognitive control under more complex activities (Michel and Koenig, 2018; Jia et al., 2021; Jia and Zeng, 2021; Takarae et al., 2022; Zhao et al., 2024).

Seven microstate classes were computed following the approach proposed in Pascual-Marqui et al. (1995). We first calculated the Global Field Power (GFP) from the pre-processed EEG data for each session (including the two resting sessions and 22 training sessions) and identified the GFP peaks corresponding to the local maxima in the GFP time series. To ensure the selection of independent and significant peaks, a minimum distance of 10 time points between peaks was applied. After identifying these GFP peaks, only the EEG data corresponding to the time points of the GFP peaks were sent to the modified k-means algorithm. We then use a modified k-means clustering algorithm, which employs a predefined cost function (detailed in Supplementary Equation S1), to generate the K clusters by minimizing the cost function. To determine the optimal microstate classes for each task of each participant, we repeat such clustering process 100 times and select the best set of clusters, namely the optimal microstate set, using the cross-validation metric which is descripted in details in the Supplementary Equation S2. This step helps address the sensitivity of k-means clustering to initial conditions, ensuring a more robust identification of microstate classes for each session (22 training sessions and two resting sessions).

In the subsequent analysis, we implemented a full permutation procedure to determine the group-level microstate classes. The full permutation process included multiple levels: determining the optimal clusters for each subject (across tasks under the same condition), each condition (across subjects), and ultimately the overall optimal clusters (across all conditions including the resting condition consisted of two resting sessions), with the clustering repeated 100 times at each level. At each iteration, we randomly selected a set of clusters from the concatenated list of clusters, and the set of clusters with the best cross-validation value was considered the optimal microstates. The resulting group-level microstate classes were referred to as global microstate classes, labeled as A, B, C, D, E, F, and G. We represented the pre-processed EEG data in the time domain by associating each time point with a specific global microstate class. To assign these labels, we computed the spatial correlation between the EEG scalp map at each time point and each of the global optimal microstate maps. The assignment criterion involved selecting the global microstate class with the highest spatial correlation value disregarding the polarities. Additionally, our analysis refrained from applying any smoothing parameters to ensure that the temporal dynamics of the generated microstate sequences remained unaltered.

Thereafter, microstate parameters were computed from the generated microstate time series based on the global optimal maps. That is, we used the obtained seven global microstates to label the EEG time series leading to 22 microstate sequences for each participant's different tasks throughout the pilot training process. The three types of microstate parameters computed for each of the global optimal microstates in this study are listed below:

- Mean microstate coverage: the fraction of the total analysis time covered by a microstate. The microstate coverage can be interpreted as the relative rather than absolute presence of a microstate.
- Mean microstate duration: the average lifespan or duration that a microstate remains stable. The microstate duration can be interpreted as the average amount of time that a set of neural generators remains synchronously active.
- Mean microstate occurrence: the average number of times that a microstate occurs per second. The mean microstate occurrence can be interpreted as the average amount of times that a set of neural generators becomes synchronously active.

### 2.5 Statistical analysis

The repeated measures analysis of variance (ANOVA) was employed to investigate the effects of microstate classes and training stages on the three types of microstate parameters under a pilot training process. For each computed EEG microstate parameter, namely coverage, occurrence, and duration, a 7 (CLASS) × 3 (STAGE) repeated measures ANOVA was applied. The two investigated within-subject factors were CLASS (microstate class A to G), and STAGE (Training, PracticeA, and PracticeB). Greenhouse-Geisser correction was applied in the case of sphericity violations. Moreover, a *post hoc* paired *t*-test was conducted between each microstate CLASS and between STAGE for multiple comparisons on the computed microstate parameters with Bonferroni correction for multiple comparisons.

The subjective evaluations of pilot trainees' aircraft control abilities were analyzed using a 5 (DIMENSION) × 3 (STAGE) repeated measures ANOVA. The two within-subject factors were DIMENSION (Performance-heading, Performance-altitude, Performance-rate climb/descent, Control-roll, Control-pitch) and STAGE (Training, PracticeA, and PracticeB). Greenhouse-Geisser correction was applied in the case of sphericity violations. A *post hoc* paired t-test was conducted between STAGE for each evaluated dimension with Bonferroni correction for multiple comparisons.

Inter-subject correlation analysis was thereafter performed to explore the associations between the EEG microstate features and pilot trainees' aircraft control performance which was subjectively evaluated by experienced training instructors. To assess the relationship between the microstate parameter metrics and the evaluation results for aircraft control abilities, a non-parametric measure was employed due to its suitability for evaluating the monotonic nature of the association between these two variables considering their inherent characteristics. In particular, we computed the Spearman correlation coefficients between the three microstate parameters (duration, occurrence, and coverage) on each microstate and the subjective evaluations of aircraft control. Concurrently, the *p-*values with Bonferroni correction for multiple comparisons were considered to assess the statistical significance of the observed correlations.

## 3 Results

### 3.1 Subjective evaluations of aircraft control abilities

The subjective evaluation of pilot trainees' aircraft control performance was conducted using a combination of qualitative and quantitative assessment. The evaluation process involved a comprehensive assessment of the training trials across three distinct aspects encompassing the quality of the dataset, a quantitative evaluation of performance in maneuvering the simulated aircraft under specific tasks, and a descriptive analysis of the actions undertaken by the trainees. To ensure an appropriate grading scale, a panel of two instructors collaborated to establish the levels of evaluation tailored to the expected performance of ab-initio candidates for each parameter. To minimize variability in assessments, a single qualified instructor was designated to evaluate the pilot trainees' control skills of the simulated aircraft.

Among the three aspects analyzed, the quality of the dataset is inherently reflected in the results obtained from the quantitative assessment of the trainees' aircraft control performance. However, the descriptive analysis provides supplementary information that can contribute to a more comprehensive understanding of the factors influencing trainees' control performance, whether positive or negative. For this research, the focus was primarily on the quantitative assessment aspect, which not only effectively reflects pilot trainees' proficiency in controlling the simulated aircraft throughout the training process, but also takes the control behavior recorded by the simulator into consideration.

The averaged subjective evaluation results are listed in Table 1 and the related p-values for paired STAGE comparisons with Bonferroni correction can be found in Table 2. The quantitative assessment encompassed five dimensions in total, consisting of three performance indicators (heading, altitude, and rate of climb/descent) and two evaluations of control management (roll and pitch), which are denoted as D1-D5 in this research as illustrated in Table 1. To be more specific, D1 corresponds to the “control-roll” dimension, D2 denotes the “performance-heading” dimension, D3 is mapped to the “control pitch” dimension, D4 represents the “performance-altitude” dimension, and D5 is associated with the “performance-rate climb/descent” dimension. Among the five dimensions under evaluation, performance-altitude (D4) was the only dimension that showed significant increases from Training to PracticeA as well as from Training to PracticeB stage (Table 2). The 5 × 3 repeated measures ANOVA on trainees' control performance statistics revealed two significant main effects of DIMENSION [*F*_(4,92)_ = 388.045, *p* = 0.000, η^2^ = 0.710] and STAGE [*F*_(2,46)_ = 31.066, *p* = 0.000, η^2^ = 0.081], as well as one significant interaction effect of DIMENSION × STAGE [*F*_(8,184)_ = 7.208, *p* = 0.000, η^2^ = 0.026]. Moreover, pilot trainees' evaluation statistics were further applied in our correlation analysis to gain insight on how the extracted EEG microstate features may relate to aircraft control levels. Given the significant main effect of DIMENSION, the five control performance dimensions were analyzed one by one in the correlation analysis.

**Table 1:** Averaged evaluation results [Mean (SE)] for pilot trainees' aircraft control performance.

**Evaluation dimension**	**Training**	**Practice-A**	**Practice-B**
D1: Control-roll	3.38 (0.082)	3.27 (0.087)	3.46 (0.066)
D2: Performance-heading	3.68 (0.066)	3.72 (0.000)	3.77 (0.065)
D3: Control-pitch	2.21 (0.110)	2.27 (0.095)	2.27 (0.124)
D4: Performance-altitude	3.13 (0.124)	3.34 (0.098)	3.50 (0.091)
D5: Performance-rate climb/descent	2.36 (0.107)	2.38 (0.121)	2.49 (0.121)

**Table 2:** *P-*values for paired STAGE comparisons on each performance evaluation dimension.

**Comparisons**	**D1**	**D2**	**D3**	**D4**	**D5**
Training vs. PracticeA	0.463	1.000	1.000	0.046*	1.000
Training vs. PracticeB	1.000	0.567	1.000	0.001*	0.942
PracticeA vs. PracticeB	0.130	1.000	1.000	0.066	0.949

* ρ ≤ 0.050.

### 3.2 EEG microstate parameters

**Figure 3:**
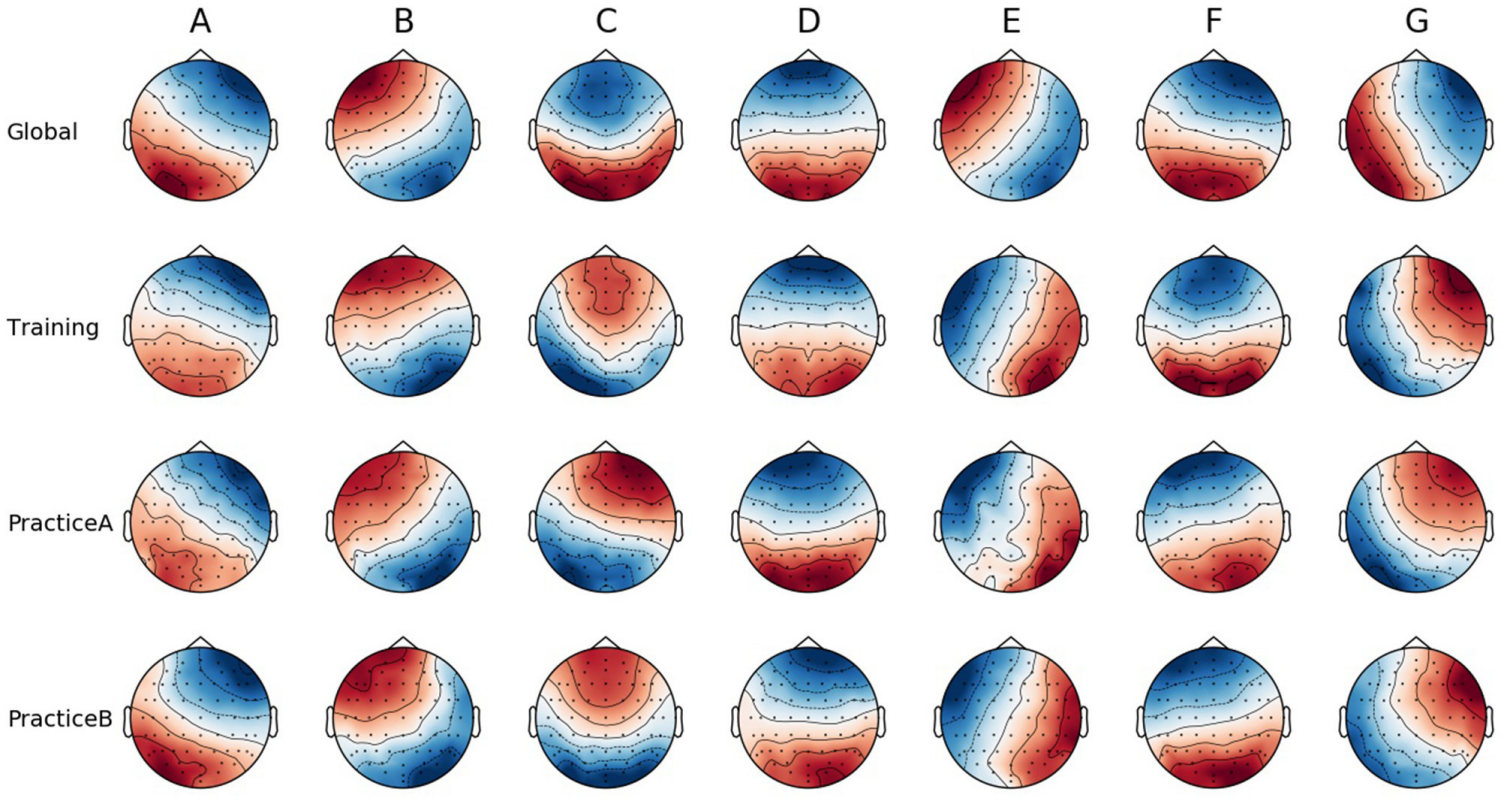
The spatial configuration of the seven microstate classes across STAGE (global) and for each training stage.

Figure 3 shows the topographic maps of seven global microstate classes across STAGE, as well as for each stage within the pilot training process, namely Training, PracticeA, and PracticeB. The seven microstate classes were labeled as A, B, C, D, E, F, and G according to Custo et al. (2017), Michel and Koenig (2018), and Jia and Zeng (2021). The seven microstate classes explained 67.127% (*SE* = 0.348) of the global variance of the original EEG topographies corresponding to peaks of GFP for Training stage, 67.894% (*SE* = 0.264) for PracticeA stage, 68.342% (*SE* = 0.301) for PracticeB stage.

**Figure 4:**
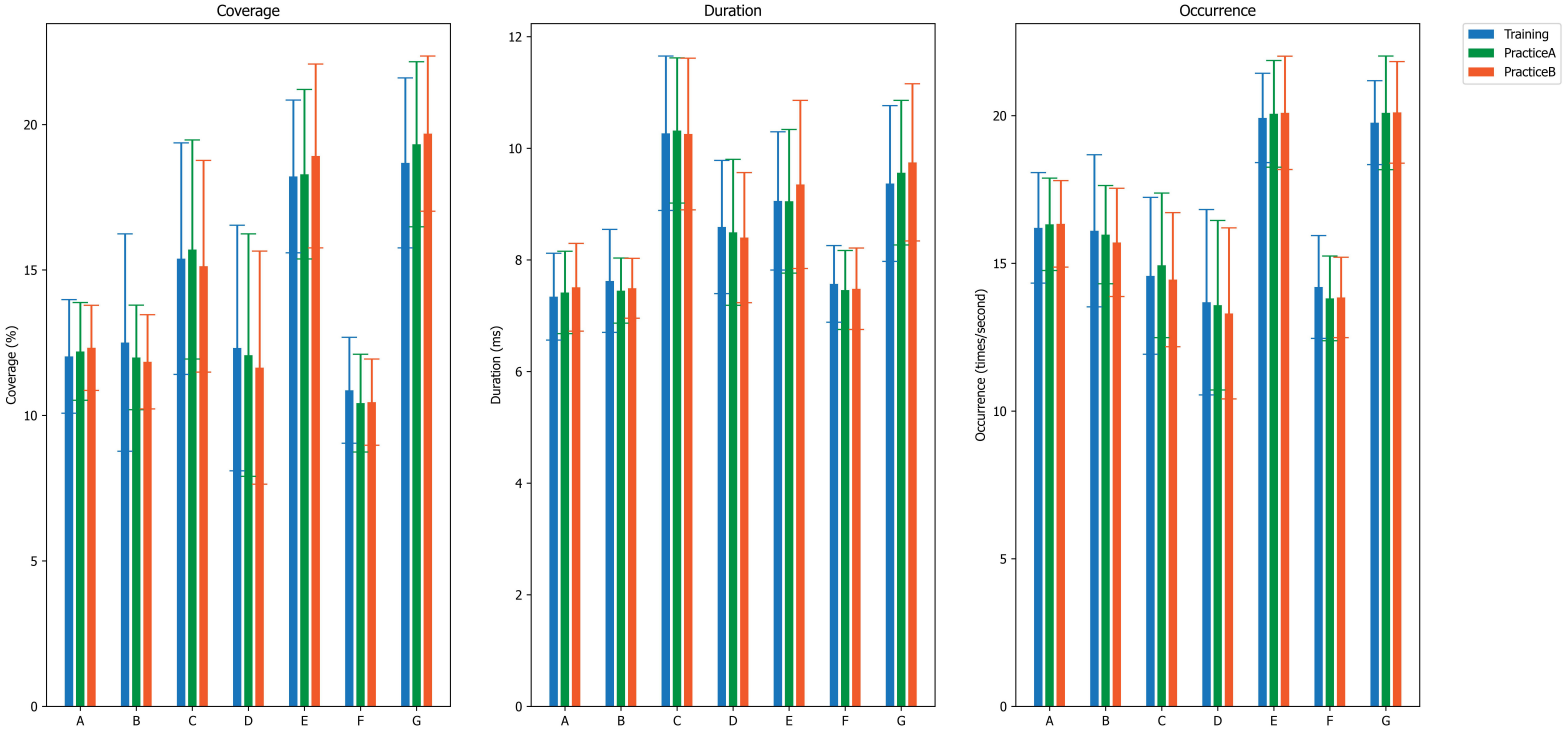
EEG microstate parameters of each microstate class during three stages: Training, PracticeA, and PracticeB.

The computed EEG microstate parameters include coverage, occurrence, and duration, as illustrated in Figure 4. As shown in Table 3, the 3 × 7 repeated measures ANOVA revealed one significant main effect of CLASS [*F*_(6,138)_ = 36.544, *p* = 0.000, η^2^ = 0.558] for microstate coverage analysis. Similarly, the 3 × 7 repeated measures ANOVA on the other two microstate parameters also revealed one significant main effect of CLASS [*F*_(6,138)_ = 56.952, *p* = 0.000, η^2^ = 0.665] for duration and one significant main effect of CLASS [*F*_(6,138)_ = 32.972, *p* = 0.000, η^2^ = 0.540] for occurrence.

**Table 3:** Effects of microstate classes on three time-domain microstate parameters under pilot training tasks.

**Parameter**	**Source**	**Num DF**	**F-value**	***P*-value**	**η^2^**
Coverage	Classes	6	36.544	0.000	0.558
	Conditions	2	0.630	0.503	0.000
	Classes × Conditions	12	1.358	0.266	0.004
Duration	Classes	6	56.952	0.000	0.665
	Conditions	2	0.607	0.524	0.000
	Classes × Conditions	12	0.934	0.419	0.002
Occurrence	Classes	6	32.972	0.000	0.540
	Conditions	2	11.500	1.000	0.000
	Classes × Conditions	12	1.192	0.317	0.004

Consistently, no significant differences were found in any of the investigated microstate parameters between different stages across seven microstate classes according to our paired *t*-test results (more details could be found in Table S8). In terms of CLASS comparisons, significant differences were observed in multiple paired comparisons between microstate classes, with effects detected in one or more microstate parameters. For example, our findings indicate that microstate class G differs significantly from microstate classes A, B, and F throughout the training process (across all three stages), with evidence observed across the three tested parameters. Detailed significance results are provided in Tables S5–S7 in the Supplementary material.

### 3.3 Correlations between expert evaluations and EEG microstate parameters

**Figure 5:**
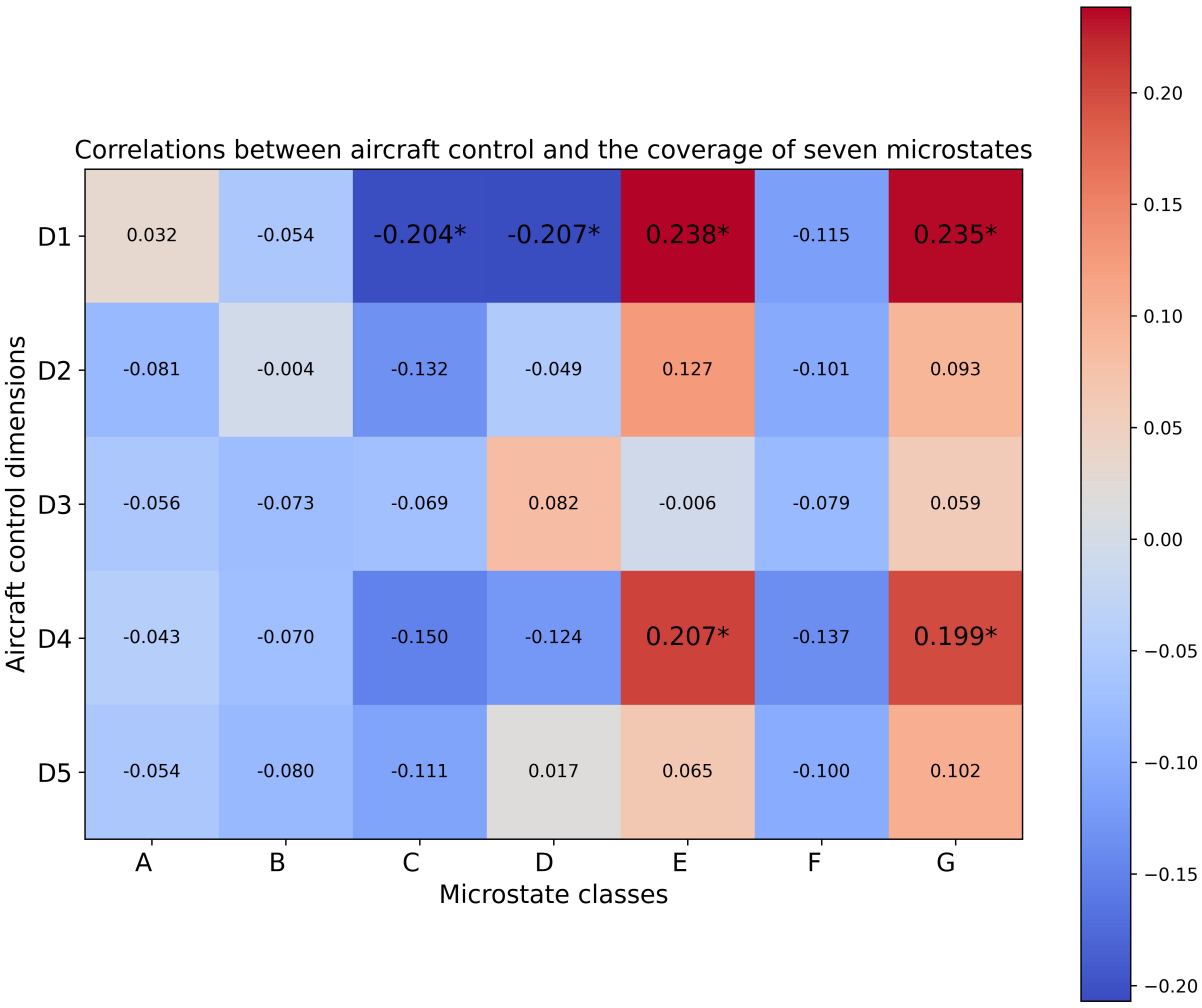
Spearman correlation coefficients between the coverage of seven microstate classes and five aircraft control dimensions. Correlations with *p*-values satisfying *p* ≤ 0.05 are annotated by ^*^.

**Figure 6:**
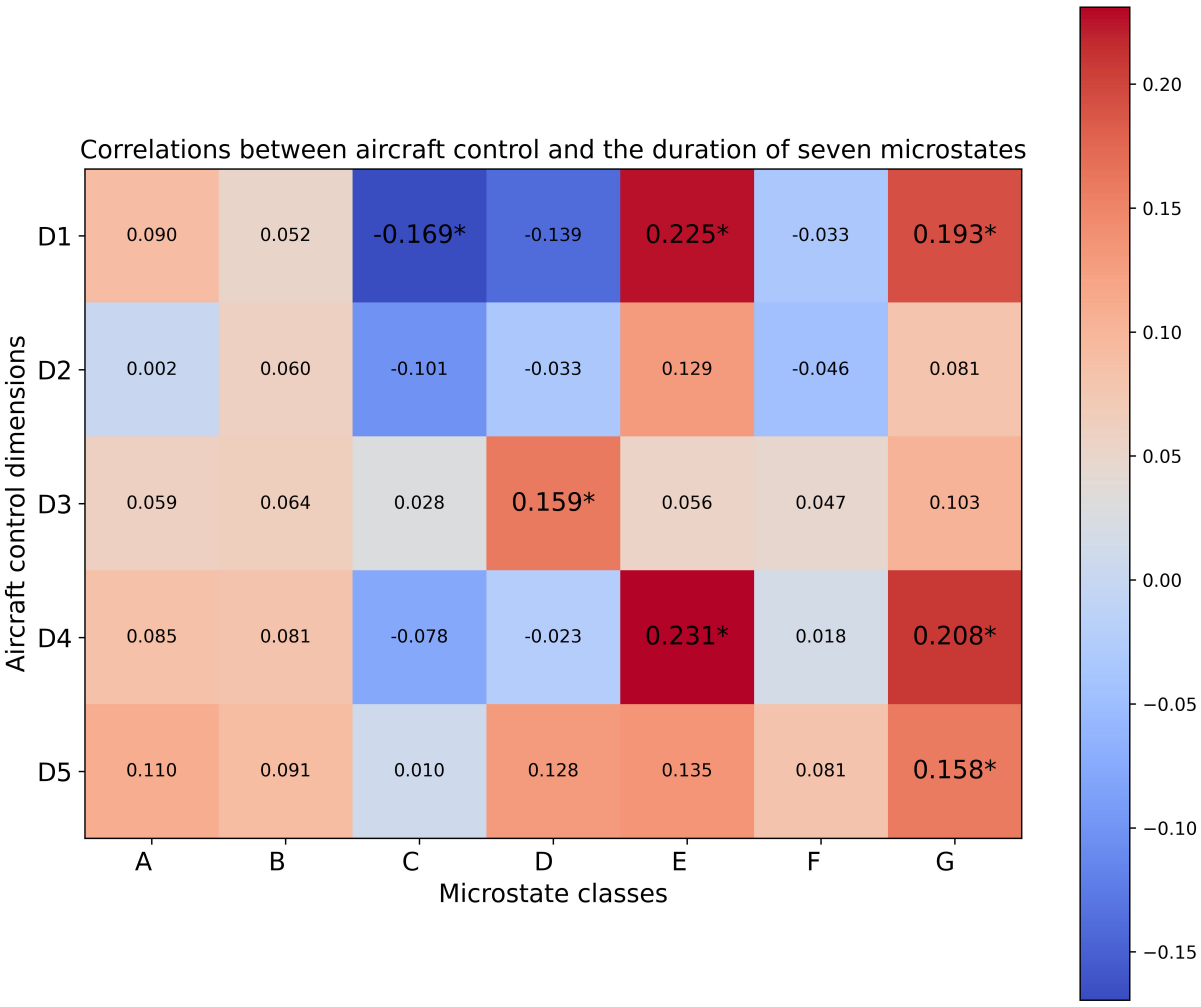
Correlation coefficients between the duration of seven microstate classes and five aircraft control dimensions. Correlations with *p-*values satisfying *p* ≤ 0.05 are annotated by ^*^.

**Figure 7:**
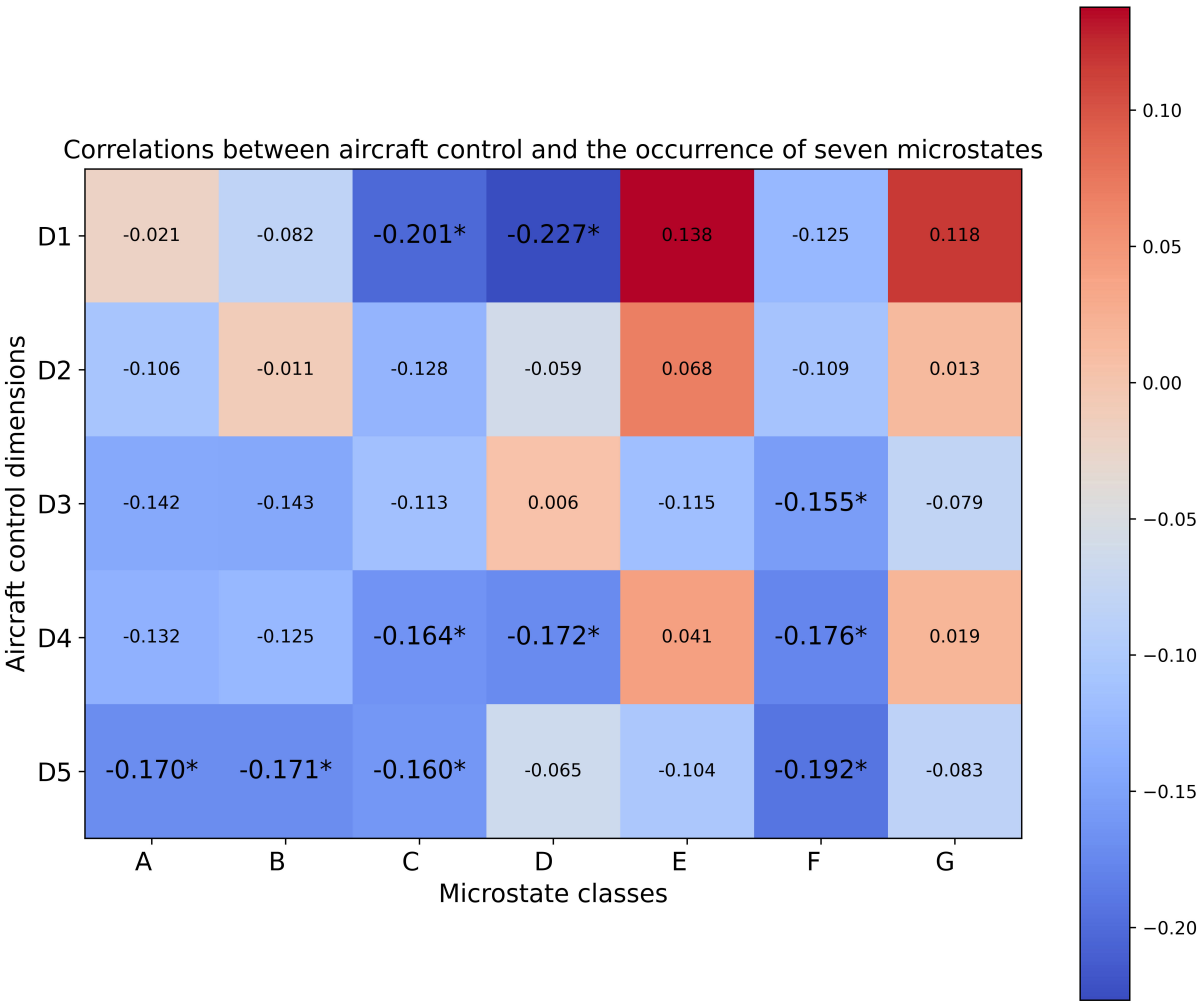
Correlation coefficients between the occurrence of seven microstate classes and five aircraft control dimensions. Correlations with *p*-values satisfying *p* ≤ 0.05 are annotated by ^*^.

As depicted in Figures 5–7, the computed Spearman correlation results were visualized using heatmaps, where positive and negative correlations were differentiated with colors. Moreover, correlations with p-values smaller than 0.05 were considered statistically significant in our analysis and were denoted with an asterisk ^*^ in Figures 5–7. The five aircraft control dimensions included in the subjective evaluations were denoted as D1 to D5, each of which captures a distinct aspect of trainees' behavior and performance on the given aircraft control tasks. The D1-D5 annotations represent the five aircraft control dimensions within the subject expert ratings described in Table 1, corresponding to the “control-roll” dimension, “performance-heading” dimension, “control pitch” dimension, “performance-altitude” dimension, and “performance-rate climb/descent” dimension respectively.

The Spearman correlation results between trainees' subjective performance valuations and the parameter features extracted from EEG microstate analysis revealed positive correlations between class G and all of the five evaluation dimensions as indicated by the coverage and duration results (Figures 5, 6). To be more precise, the coverage of microstate class G showed significant positive correlations with evaluation dimensions including D1 and D4, whereas the duration of microstate class G was significantly positively correlated with D1, D4, and D5. Even though the occurrence results of class G didn't show any significant correlations (Figure 7), the positive correlations between class G and the evaluation dimensions D1 and D4 were consistently supported across the temporal parameters. Moreover, the coverage and duration of microstate class E both showed significant positive correlations with two subjectively evaluated dimensions, D1 and D4 (Figures 5, 6), and the occurrence results also supported the positive correlations between class E with these two dimensions with non-significant correlations (Figure 7). As for class D, the only significant positive correlation was observed between the duration parameter and evaluation dimension D3 (Figure 6). Meanwhile, the duration of microstate class A and class B showed consistent positive correlations across the evaluated dimensions though none of those correlations were significant (Figure 6).

Besides the positive correlations discussed above, the correlation results showed significant negative correlations between the occurrence of microstate class C and dimensions D1, D4, and D5, as illustrated in Figure 7. Consistently, significant negative correlations were also observed between dimension D1 and the other two parameters of class C, namely coverage and duration (Figures 5, 6). Significant negative correlations were also observed between the occurrence of microstate class F and dimensions D3, D4 and D5 (Figure 7). Significant negative correlations between the coverage of microstate classes C and F were observed with D1 and D5 (Figure 5). In the meantime, the correlation results indicated by the two parameters, coverage and occurrence, revealed consistant negative correlations for both class C and class F across the evaluated dimensions. Moreover, we observed consistent negative correlations between two parameters (coverage and occurence)of microstate classes A and B with most of the aircraft control dimensions, though most of which were non-significant (Figures 5, 7). The occurrence of both microstates class A and class B retained significant negative correlations with the evaluation dimension D5.

Among the five aircraft control dimensions included in the subjective evaluations, D1 and D4 exhibited noticeable consistency in the reported significant correlations across the three types of microstate parameters extracted from seven microstate classes (Figures 5–7). In particular, the subjective evaluation dimension D1 exhibited significant negative correlations with microstate class C in all three types of microstate parameters, whereas significant positive correlations were observed between D1 with microstate classes D and G in two out of three parameters, namely coverage and duration (Figures 5, 6). Also, the parameters coverage and occurrence of microstate class D indicated significant negative correlations with dimension D1 (Figures 5, 7). Moreover, the subjective evaluation dimension D4 exhibited significant positive correlations with microstate classes E and G as indicated in the parameters coverage and duration (Figures 5, 6).

Moreover, the correlation results also revealed significant correlations between certain dimensions covered in the subjective evaluations and one or two parameters of a specific microstate class. More detailed information can be found in (Figures 5–7).

## 4 Discussions

### 4.1 Does brainwave dynamics correlate with subjective evaluations of aircraft control?

Our research revealed significant correlations between EEG microstate parameters and expert evaluations of aircraft control performance in pilot trainees. Both positive and negative correlations were observed, providing insights into the cognitive functions involved in pilot training. Specifically, microstate classes E and G had significant positive correlations with most subjective control performance dimensions, while microstate classes C and F showed significant negative correlations. These findings suggest that different cognitive processes, as indicated by various microstate classes, play distinct roles in enhancing or impairing control capabilities during pilot training.

The positive correlation between microstate class E and pilot trainees' subjective performance evaluations suggests that the activation and enhanced engagement of this microstate may be a key indicator of effective pilot training. The increased presence of microstate class E indicates better attentional allocation and integration of sensory inputs, which are essential for achieving expertise in aircraft control. This is consistent with previous research linking microstate class E to attentional processes and perceptual integration (Khanna et al., 2015). Attentional mechanisms are crucial in monitoring and responding to multiple sources of information during complex tasks like aircraft control (Larson and Clayson, 2011; Valéry et al., 2017; Holmes et al., 2014). Thus, enhanced engagement of the neural configuration captured by microstate class E likely facilitates efficient information processing and motor coordination necessary for successful aircraft control. Similarly, the positive correlation between microstate class G and better aircraft control abilities, as reflected in subjective evaluations, highlights the importance of cognitive processes associated with decision-making and executive control in aviation. Microstate class G has been linked to higher-order cognitive functions such as working memory and cognitive flexibility (Van de Ville et al., 2010). Effective aircraft control demands continuous monitoring of various factors, swift decision-making, and adaptive responses to changing situations. Jia et al. (2021) found that microstate class G was more prevalent in designers engaged in internally guided decision-making than in externally guided decision-making tasks. This suggests that the enhanced engagement of microstate class G reflects the recruitment of executive control processes, which facilitate efficient information processing, strategic planning, and adaptive behavior, ultimately contributing to superior aircraft control performance.

The negative correlation between microstate class C's parameters and pilot trainees' subjective evaluations may indicate the involvement of cognitive control processes in aircraft control tasks. Cognitive control processes, which involve the regulation and coordination of cognitive functions, such as attention, inhibition, and working memory, play a critical role in complex tasks like aircraft control (Niendam et al., 2012; Miller and Cohen, 2001). Microstate class C, a distinct pattern of synchronous neural activity observed in EEG recordings, has been reported for both positive and negative correlations with cognitive control mechanisms as reviewed in Michel and Koenig (2018). For example, research has shown that microstate class C is related to the engagement of cognitive control networks including the prefrontal cortex and anterior cingulate cortex (Khanna et al., 2015; Milz et al., 2016). Microstate C has also been found to reflect activities in the default mode network (DMN) supported by EEG and fMRI evidence (Xu et al., 2016; Seitzman et al., 2017; Bréchet et al., 2019). From this standpoint, our results could also support the notion that microstate class C is negatively correlated to control skills, which was consistent with the studies suggesting this specific microstate's role in reflecting activities in the default mode network (DMN). Furthermore, the negative correlation between microstate class F and pilot trainees' subjective evaluations further emphasizes the role of the DMN in enhanced aircraft control under the context of pilot training. Microstate class F has been associated with the DMN, primarily involving regions such as the medial prefrontal cortex, posterior cingulate cortex, and angular gyrus (Khanna et al., 2015; Musso et al., 2010; Van de Ville et al., 2010). The DMN is a network of brain regions that is active during internally focused, self-referential, and mind-wandering states, and it becomes suppressed during goal-directed tasks (Dohmatob et al., 2020; Carhart-Harris and Friston, 2010). Our results may indicate the negative associations between DMN and better aircraft control performance.

In summary, the aforementioned correlations observed between certain microstate classes and pilot trainees' subjective evaluations of their training performance provide insights into the neural underpinnings of skilled aircraft control. In particular, the observed negative correlations between the parameters of microstate class C and class F with subjective performance evaluations suggest that an inefficient suppression or interference from the DMN during task execution might impede trainees' ability to maintain focused attention and cognitive resources on the aviation control tasks at hand. Our findings align with previous research demonstrating that excessive activation or inefficient suppression of the DMN is associated with poorer task performance across various domains (Anticevic et al., 2012; Duan et al., 2012; Harrison et al., 2011). In the meantime, we also observed the positive correlations between the parameters of microstate class E and class G with pilot trainees' subjective performance evaluations. As indicated by our experimental results, there might exist positive associations between aircraft control expertise and several cognitive aspects like attentional processes, perceptual integration, working memory, cognitive flexibility, and executive control, which could be considered to improve the current pilot training efficiency. Further research is needed to elucidate the precise mechanisms through which these microstates influence pilot performance and explore their potential applications in training and optimizing aviation expertise.

### 4.2 What added value does brainwave dynamics bring for a more accurate and comprehensive pilot performance evaluation?

The integration of neuroscientific evidence from EEG analysis allows for a detailed understanding, confirmation, and calibration of the interrelationships among various assessment dimensions included in current evaluation systems. For instance, D3 (control-pitch) and D5 (performance-rate climb/descent) show positive correlations with the duration parameter across the seven microstate classes whereas these two dimensions also indicated consistent negative correlations with the occurrence parameter across most of the computed microstates. This neuroscientific evidence aligns with the inherently connected relationship between D3 (pitch control) and D5 (rate of climb/descent) from the instructors' standpoint where D3 control enables the performance of D5, and could serve as the validation for each other. To achieve a good rate of climb or descent, effective pitch control is essential. This interrelationship can be seen as analogous to the relationship between a root cause (D3) and its symptom (D5). Good pitch control is fundamental to ensuring a proper rate of climb or descent, which is why these two elements are deeply interrelated for a practical understanding of vertical control in aviation. In the meantime, D1 (control-roll) and D4 (performance-altitude) exhibit significant correlations with several microstates across parameters, suggesting that those two dimensions could serve as holistic measures of trainees' aircraft control abilities. Dimensions D1 and D4 were not only involved in the aforementioned significant positive correlations with microstate classes like E and G, but also indicated significant negative correlations with microstate classes C and F as discussed. Emphasizing such dimensions in future performance evaluations may provide a more accurate and comprehensive assessment of a pilot's cognitive and control capabilities.

Moreover, advanced EEG analysis techniques provide an objective reflection of trainees' cognitive states, which could be integrated into the evaluation protocol to improve accuracy and reliability. This precision ensures that training feedback incorporates objective data alongside traditional performance metrics, providing a richer understanding of trainee behavior and performance. For example, we may also infer from the microstate-based similarities observed between dimensions D3 and D5 that both dimensions reflect the continuous involvement of visual and attentional resources under flight tasks alongside the aforementioned alignment. Evidence from EEG microstates reveals the shared cognitive and neural mechanisms underlying these dimensions, adding extra angles to instructors' understanding of the trainees' cognitive states during the training process. The involvements of microstate classes A and B highlight the visual monitoring and information processing consistently required for both pitch control and assessing the rate of climb or descent during flight tasks. Also, the activation of microstate class G underscores the importance of attentional control in executing precise pitch adjustments and maintaining the desired vertical trajectory. Another example is the significant positive correlations observed between microstate class G and multiple dimensions (D1, D4 in both coverage and duration, and D5 only in duration), indicating its critical role in reflecting cognitive abilities under aircraft control tasks. Further investigations into this microstate could contribute to a better understanding of a pilot's cognitive changes throughout the training program. Such a holistic approach addresses both cognitive and physical aspects of performance, leading to more effective training outcomes.

Our experimental results provide insights into integrating advanced analytical techniques with subjective and objective evaluations for a more comprehensive, efficient, and effective approach to assessing pilots' aircraft control abilities. These scores can be weighted to reflect the relative importance of each dimension based on empirical data from microstate correlations, while subjective evaluations and feedback from instructors remain crucial and should be integrated recursively with objective assessments. This ensures a holistic approach, as instructors can provide nuanced insights that may not be captured through objective measures alone. The continuous interplay between subjective and objective evaluations enriches the training process, offering a complete understanding of a trainee's performance and cognitive state. Following our discussions on the consistent correlations between D1 and D4 with various microstate classes, we observed similarities between three of the evaluated performance dimensions, namely D1, D2, and D4 considering both significant and non-significant correlations. Therefore, grouping some of these dimensions under the umbrella of vertical control seems logical from both an evaluative and a neurocognitive standpoint, as they share common underlying cognitive processes. However, breaking them down into separate components provides the necessary granularity to assess each aspect individually, thereby facilitating a more comprehensive evaluation and targeted interventions. That is to say, combining and balancing subjective feedback and objective measures is key to achieving greater efficiency and effectiveness in pilot training, allowing for more precise, adaptable training programs that address individual needs while leveraging empirical data to optimize learning outcomes. We aim to optimize the pilot evaluation protocol by integrating advanced analytical techniques to ensure a more targeted, accurate, and holistic approach to evaluating pilots' aircraft control skills.

### 4.3 How to enable adaptive and personalized training by utilizing brainwave dynamics?

The integration of EEG data allows for detailed feedback on specific cognitive functions associated with different training tasks, benefiting both pilot instructors and trainees by providing a clearer understanding of trainees' cognitive control, engagement, and workload. By combining objective and subjective feedback, training programs can be tailored to address individual trainees' specific cognitive status and aircraft control skills. For example, trainees exhibiting higher levels of microstate class C can receive additional training focused on maintaining engagement and increasing cognitive control abilities. In this way, the EEG-enabled individualized approach ensures that training programs are adjusted to each trainee's cognitive states and control levels, enhancing the overall training effect. Personalized training plans and materials can also help minimize cognitive overload and improve training effectiveness.

Additionally, we aim to incorporate real-time EEG microstate analysis into the evaluation protocol in our future work, allowing continuous monitoring of trainees' cognitive states throughout the training process. This integration can provide immediate feedback and enable dynamic adjustments to training programs based on real-time data. By adapting training programs to individual trainees' cognitive profiles, training efficiency, and effectiveness can be enhanced without overwhelming instructors with extra task loads. When training instructors detect EEG-based signs of cognitive overload or disengagement in their trainees, they can provide personalized and timely interventions to improve learning efficiency. Furthermore, continuous validation and refinement of training protocols based on empirical EEG data ensure the effectiveness of the training materials and program settings. Regular updates based on the latest research findings can further enhance the accuracy and reliability of evaluations, leading to a more efficient and effective training process.

In summary, the research findings presented in this project highlight the potential for improving pilot training processes by integrating advanced EEG analytical techniques into pilot evaluations. These techniques can objectively reflect trainees' cognitive states, enhancing the accuracy and reliability of evaluations. Incorporating real-time EEG microstate analysis for continuous monitoring and adaptive training programs can enable personalized training and timely interventions. Considering the practical aspects of obtaining EEG information and deriving microstates under a pilot training process, we will continue to explore less invasive alternatives while current EEG methodologies are indeed intrusive. Our future research will continue to refine the microstate features to develop a metric that is more readable and user-friendly for instructors. This will facilitate easier integration into existing training protocols without adding significant operational complexity, ultimately improving the effectiveness and efficiency of pilot training.

## 5 Limitations and future work

The present research has some limitations that need to be carefully considered in our future work. Firstly, one limitation of this study is the lack of behavioral analysis of trainees' learning behaviors recorded by cameras. This limitation arises mainly due to the subjectivity involved in behavior analysis. Recognizing the importance of integrating such behavioral analysis into our research, we consider it as one of our future directions to complement the results obtained from physiological measures and subjective assessments. Another limitation lies in the lack of direct analysis of the objective results recorded by the simulator, although the objective aspect has been considered for the generation of expert evaluations. Our expert ratings were based on time-series visualization of aircraft heading, angle of bank, altitude, pitch angle, vertical speed, and control movements. And results presented in Jennings et al. (2024) also demonstrated that the objective performance metrics from our dataset are proportional to those objective expert evaluations. As this research highlights the common utilization of subjective evaluations in practical pilot training processes, we will include the analysis of objective performances in our future work without distracting readers' attention from the EEG-based insight into current pilot training protocols. Additionally, the study primarily focuses on pilot trainees, which may limit the applicability of the findings to experienced pilots or aviation professionals. Therefore, we plan to expand the dataset in our future research by including larger datasets and more diverse participant groups, including both novices and experienced pilots, to enhance the generalizability of the findings. However, this may enhance the potential generalizability of the current study to other training or learning processes. Furthermore, it is worth noting that the research primarily relies on EEG-based analysis, which offers a high temporal resolution but may lack spatial specificity. In our future work, we will continue to incorporate other techniques such as ECG and GSR through wearable devices. These methods will allow for more practical and naturalistic assessments of cognitive control, complementing the advantages of EEG in real-world pilot training scenarios as reported in Darvishi-Bayazi et al. (2023) and Ruiz-Segura et al. (2024).

## 6 Conclusion

In conclusion, this research investigates the correlations between pilot trainees' subjective performance evaluations and their brainwave dynamics during a pilot training process, as revealed through EEG microstate analysis. The experimental results showed significant associations between the temporal parameters of a few specific microstates and the aircraft control performance dimensions included in the subjective evaluations, highlighting the multidimensional nature of aircraft control proficiency. To be more precise, the observed positive correlations between subjective performance evaluations with microstate class E and class G reflected the involvement of attentional processes, perceptual integration, working memory, and executive control in skilled aircraft control in the context of pilot training. On the other hand, the observed negative correlations between subjective performance evaluations with microstate class C and class F suggest the associations between decreased default mode network (DMN) in pilot trainees and their aircraft control abilities expected to be achieved at the end of a pilot training process. These results provide neurophysiological markers that could be employed in designing targeted training programs and interventions to optimize the training efficiency in aviation control skills. Additionally, the research highlights the potential of using EEG microstate analysis as a non-invasive and cost-effective tool to objectively assess trainees' cognitive states, enhancing the accuracy and reliability of evaluations. Overall, our research findings could not only provide implications for cognitive neuroscience and human factors in aviation expertise but also inspire further exploration and applications in improving the efficiency and effectiveness of pilot training programs.

## Data Availability

The raw data supporting the conclusions of this article will be made available by the authors, without undue reservation.
