# Supplementary material for: Correlations of pilot trainees' brainwave dynamics with subjective performance evaluations: insights from EEG microstate analysis
Source: Front Neuroergon. 2025 Mar 5;6:1472693. doi: 10.3389/fnrgo.2025.1472693 (PMC11919915; doi:10.3389/fnrgo.2025.1472693)
Supplement: Supplementary file 1 [file Data_Sheet_1.pdf]

I. Demographic information details

Table S1 Demographic characteristics of he study participants (N=24)

Characteristic	Category	Number of Participants (n)	Percentage
**Age Group**	21-30 years	16	66.7%
	31-40 years	7	29.2%
	41-50 years	1	4.2%
**Gender**	Female	13	54.2%
	Male	11	45.8%
**Education Level**	High school diploma	3	12.5%
	Bachelor's degree	9	37.5%
	Master's degree	12	50.0%
**Handedness**	Right-handed	22	91.7%
	Left-handed	2	8.3%
**Previous experience**	Experience with any type of flight simulator	7	29.2%
	No Prior Experience	17	70.8%

Data Collection Procedure:

- **Demographic Questionnaire:** Before commencing the experimental tasks, participants completed a questionnaire capturing demographic information, including age, gender, education level, handedness, and prior flight experience.
- **Confidentiality Assurance:** Participants were assured that all personal data would be kept confidential and used solely for research purposes.

Ethical Considerations:

- The experimental protocol was approved by both the Concordia Human Research Ethics Committee and the research ethics board of the National Research Council Canada.
- Participants were informed of their right to withdraw from the study at any time without penalty.

This section provides detailed demographic information about the participants involved in the study, ensuring transparency and enabling readers to understand the composition of the sample. It adheres to ethical standards by maintaining participant confidentiality and providing all necessary information for reproducibility and further analysis.

II. Pre-processing details on bad channels and components

Table S2 Total number of bad channels identified and removed based on the applied criteria

Session	1	2	3	4	5	6	7	8	9	10	11	12	13	14	15	16	17	18	19	20	21	22
P01	12	7	13	10	11	13	9	1	1	1	3	0	0	1	1	0	1	1	1	1	2	2
P02	3	3	2	2	2	2	3	1	5	4	2	2	2	2	3	1	1	2	0	3	1	1
P03	5	5	4	4	3	4	4	6	5	4	4	4	3	4	6	4	6	5	4	6	4	3
P04	3	5	6	4	3	7	7	3	6	3	3	3	1	11	3	3	4	2	6	5	4	2
P05	7	7	10	6	4	5	2	3	4	2	10	7	1	7	3	6	3	7	5	4	3	6
P06	0	7	1	3	6	0	1	0	0	2	0	0	0	1	0	8	4	9	6	5	1	5
P07	1	2	2	4	3	1	4	3	2	7	2	1	2	2	4	5	0	5	6	0	5	4
P08	7	5	3	7	4	8	6	8	6	4	8	4	2	6	8	6	6	6	6	4	7	3
P09	2	1	5	4	0	3	0	1	1	8	1	1	3	0	1	4	5	2	2	1	2	3
P10	7	4	7	6	7	5	6	8	3	6	4	2	8	7	4	9	2	6	0	1	1	3
P11	3	4	7	6	6	5	5	5	1	3	4	6	3	5	5	2	5	3	7	7	3	1
P12	5	3	5	4	5	4	5	7	4	6	5	2	4	4	5	5	4	2	4	3	5	4
P13	1	1	1	2	1	2	1	0	0	0	1	0	0	0	0	3	3	3	3	3	5	6
P14	6	5	3	4	2	6	2	3	5	8	5	2	8	2	5	2	5	2	5	4	2	2
P15	4	1	5	5	1	2	3	4	2	6	5	5	8	8	6	5	12	10	1	8	4	3
P16	2	1	3	3	1	4	1	3	1	1	1	1	2	4	1	2	1	1	5	3	5	2
P17	7	7	2	3	4	4	3	6	4	3	3	3	6	5	5	8	5	4	2	6	6	2
P18	5	1	1	1	1	1	1	0	0	7	0	2	1	2	8	5	8	1	1	1	1	1
P19	0	4	2	2	1	4	3	5	8	2	8	2	3	1	5	2	5	6	4	2	6	2
P20	8	6	11	9	7	10	6	4	3	6	3	4	5	3	1	4	5	0	2	3	2	6
P21	6	9	10	8	4	7	11	6	4	7	8	6	5	7	6	4	4	9	2	7	5	7
P22	2	4	1	5	0	1	2	3	0	3	2	7	3	1	3	7	0	5	6	7	2	7
P23	4	1	5	4	2	2	3	3	4	5	5	3	3	4	2	4	2	3	6	1	7	1
P24	3	2	2	3	2	7	1	5	3	2	2	1	2	1	4	4	2	1	5	4	8	1

Table S3 Percentage of remaining components after artifact removal using MARA

Session	1	2	3	4	5	6	7	8	9	10	11	12	13	14	15	16	17	18	19	20	21	22
P01	34.4	39.1	37.5	39.1	34.4	29.7	35.9	50	51.6	56.3	45.3	54.7	42.2	46.9	46.9	54.7	57.8	56.3	54.7	54.7	51.6	39.1
P02	54.7	53.1	65.6	56.3	64.1	56.3	65.6	54.7	60.9	59.4	57.8	64.1	57.8	59.4	56.3	62.5	57.8	46.9	59.4	60.9	50	67.2
P03	73.4	75	57.8	67.2	68.8	62.5	70.3	71.9	73.4	70.3	67.2	67.2	62.5	64.1	68.8	71.9	70.3	60.9	64.1	62.5	64.1	60.9
P04	57.8	56.3	48.4	56.3	53.1	57.8	50	46.9	57.8	60.9	62.5	56.3	68.8	51.6	60.9	56.3	53.1	64.1	60.9	62.5	60.9	62.5
P05	32.8	21.9	25	23.4	31.3	31.3	21.9	15.6	32.8	26.6	45.3	43.8	23.4	39.1	21.9	43.8	17.2	23.4	32.8	32.8	17.2	50
P06	64.1	59.4	73.4	67.2	67.2	65.6	54.7	70.3	64.1	60.9	57.8	59.4	57.8	53.1	60.9	70.3	68.8	60.9	64.1	64.1	57.8	59.4
P07	25	21.9	29.7	28.1	31.3	37.5	34.4	39.1	50	62.5	40.6	32.8	45.3	31.3	56.3	50	42.2	51.6	54.7	39.1	42.2	39.1
P08	73.4	75	78.1	67.2	79.7	76.6	62.5	76.6	78.1	84.4	76.6	84.4	71.9	79.7	76.6	82.8	78.1	75	87.5	79.7	79.7	64.1
P09	62.5	59.4	46.9	71.9	51.6	57.8	57.8	56.3	46.9	65.6	57.8	40.6	59.4	62.5	67.2	67.2	60.9	62.5	53.1	53.1	65.6	56.3
P10	56.3	42.2	64.1	57.8	67.2	50	62.5	65.6	42.2	54.7	43.8	35.9	62.5	54.7	64.1	71.9	64.1	62.5	79.7	71.9	60.9	64.1
P11	25	67.2	59.4	71.9	53.1	54.7	39.1	62.5	51.6	34.4	59.4	57.8	70.3	75	70.3	57.8	62.5	68.8	64.1	64.1	59.4	65.6
P12	48.4	56.3	48.4	54.7	60.9	56.3	68.8	59.4	59.4	62.5	56.3	53.1	54.7	45.3	68.8	67.2	60.9	40.6	71.9	67.2	60.9	53.1
P13	64.1	73.4	68.8	64.1	71.9	65.6	68.8	75	78.1	81.3	78.1	78.1	85.9	85.9	78.1	84.4	75	75	73.4	79.7	73.4	84.4
P14	35.9	46.9	50	50	59.4	45.3	53.1	59.4	54.7	57.8	48.4	54.7	54.7	53.1	62.5	56.3	65.6	48.4	56.3	59.4	64.1	37.5
P15	75	85.9	71.9	65.6	79.7	79.7	73.4	73.4	76.6	73.4	67.2	76.6	65.6	73.4	76.6	78.1	70.3	68.8	75	67.2	81.3	73.4
P16	48.4	37.5	48.4	43.8	40.6	45.3	42.2	46.9	51.6	45.3	48.4	40.6	42.2	51.6	57.8	59.4	42.2	39.1	51.6	42.2	56.3	53.1
P17	56.3	51.6	60.9	56.3	53.1	56.3	57.8	48.4	48.4	35.9	45.3	48.4	59.4	50	48.4	57.8	57.8	51.6	46.9	50	56.3	67.2

P18	50	50	56.3	57.8	57.8	56.3	45.3	73.4	65.6	60.9	64.1	54.7	53.1	48.4	57.8	40.6	48.4	53.1	59.4	48.4	62.5	57.8
P19	56.3	51.6	54.7	42.2	37.5	60.9	64.1	64.1	62.5	39.1	57.8	46.9	48.4	54.7	53.1	40.6	62.5	65.6	56.3	32.8	56.3	45.3
P20	60.9	42.2	50	50	50	50	46.9	70.3	67.2	51.6	51.6	50	51.6	60.9	57.8	54.7	60.9	65.6	60.9	57.8	51.6	54.7
P21	54.7	45.3	60.9	50	59.4	68.8	48.4	67.2	57.8	54.7	64.1	71.9	64.1	64.1	62.5	62.5	42.2	65.6	78.1	71.9	78.1	62.5
P22	60.9	59.4	37.5	65.6	42.2	54.7	57.8	60.9	48.4	53.1	46.9	43.8	46.9	40.6	37.5	48.4	59.4	50	48.4	50	48.4	53.1
P23	65.6	64.1	62.5	62.5	64.1	59.4	62.5	75	56.3	59.4	62.5	64.1	68.8	56.3	62.5	62.5	60.9	56.3	59.4	53.1	54.7	53.1
P24	29.7	35.9	25	26.6	31.3	34.4	45.3	35.9	35.9	26.6	31.3	32.8	34.4	25	28.1	40.6	32.8	26.6	35.9	43.8	40.6	34.4

Table S4 Mean and standard deviation of each participant's percentage of retained components

	Mean	STD
P01	46.0	8.53
P02	58.7	5.00
P03	67.1	4.66
P04	57.5	5.32
P05	29.7	9.60
P06	62.8	5.20
P07	40.2	10.45
P08	76.7	6.05
P09	58.3	7.36
P10	59.0	10.60
P11	58.8	12.12
P12	58.0	7.90
P13	75.6	6.49
P14	53.3	7.49
P15	74.0	5.13
P16	47.0	6.05
P17	52.9	6.44
P18	55.5	7.27
P19	52.4	9.38
P20	55.3	6.90
P21	61.6	9.40
P22	50.6	7.63
P23	61.2	5.04
P24	33.3	5.69

III. Equations used in the computation of microstates

Equation S1: Cost Function for k-Means Clustering

The modified k-means clustering algorithm employed in this study uses the following cost function to generate K clusters by minimizing the spatial variance across the EEG data at the GFP peaks:

$$F = \frac{1}{N_T(N_S - 1)} \sum_{t=1}^{N_T} \left\| V_t - \sum_{k=1}^{N_K} a_{kt} \Gamma_k \right\|^2 \qquad \text{(Equation S1)}$$

Where:

- N_T: Sample length.
- N_S: Number of electrodes.
- V_t: $N_S \times 1$ vector containing the electric potential at time point t.
- Γ_k : $N_S \times 1$ normalized vector representing the topography of the k-th microstate class.
- a_{kt} : Intensity of the k-th microstate class at time point t.

The algorithm minimizes F by iteratively adjusting Γ_k and a_{kt} until convergence, ensuring that the clustering process optimally represents the spatial configuration of the EEG data.

Equation S2: Cross-Validation Metric for Microstate Clustering

To address the sensitivity of k-means clustering to initial conditions, the clustering process was repeated 100 times for each task of each participant. The optimal set of microstate classes was selected based on the following cross-validation metric:

$$CV = \frac{\sum_{t=1}^{N_T} (V_t' \cdot V_t - (V_t' \cdot \Gamma_k)^2)}{N_T(N_S - 1)} \cdot \left(\frac{N_S - 1}{N_S - 1 - N_K}\right)^2 \qquad \text{(Equation S2)}$$

Where:

- N_K: Number of microstate classes.
- N_T: Sample length.
- N_S: Number of electrodes.
- V_t: $N_S \times 1$ vector containing the electric potential at time point t.
- Γ_k : $N_S \times 1$ normalized vector representing the topography of the k-th microstate class.

IV. P-values for CLASS and STAGE comparisons on microstate parameters

Table S5 Coverage results after Bonferroni correction for paired CLASS comparisons at each stage (p<0.05 highlighted)

STAGE	Training							PracticeA							PracticeB						
Class comparison	A	B	C	D	E	F	G	A	B	C	D	E	F	G	A	B	C	D	E	F	G
A	1,00	1,00	1,00	0,02	0,00	1,00	0,00	1,00	1,00	1,00	0,02	0,00	0,05	0,00	1,00	1,00	1,00	0,08	0,00	0,00	0,00
B	1,00	1,00	1,00	0,79	0,00	0,32	0,00	1,00	1,00	1,00	0,01	0,00	0,02	0,00	1,00	1,00	1,00	0,02	0,00	0,09	0,00
C	1,00	1,00	1,00	0,89	0,00	1,00	0,00	1,00	1,00	1,00	0,17	0,00	1,00	0,00	1,00	1,00	1,00	0,16	0,00	1,00	0,00
D	0,02	0,79	0,89	1,00	0,53	0,00	0,04	0,02	0,01	0,17	1,00	0,97	0,00	0,08	0,08	0,02	0,16	1,00	0,13	0,00	0,01
E	0,00	0,00	0,00	0,53	1,00	0,00	1,00	0,00	0,00	0,00	0,97	1,00	0,00	0,59	0,00	0,00	0,00	0,13	1,00	0,00	1,00
F	1,00	0,32	1,00	0,00	0,00	1,00	0,00	0,05	0,02	1,00	0,00	0,00	1,00	0,00	0,00	0,09	1,00	0,00	0,00	1,00	0,00
G	0,00	0,00	0,00	0,04	1,00	0,00	1,00	0,00	0,00	0,00	0,08	0,59	0,00	1,00	0,00	0,00	0,00	0,01	1,00	0,00	1,00

Table S6 Duration results after Bonferroni correction for paired CLASS comparisons at each stage(p<0.05 highlighted)

STAGE	Training							PracticeA							PracticeB						
Class comparison	A	B	C	D	E	F	G	A	B	C	D	E	F	G	A	B	C	D	E	F	G
A	1,00	1,00	0,00	0,00	0,00	1,00	0,00	1,00	1,00	0,03	0,00	0,00	1,00	0,00	1,00	1,00	0,04	0,00	0,00	1,00	0,00
B	1,00	1,00	0,20	0,00	0,00	1,00	0,00	1,00	1,00	0,02	0,00	0,00	1,00	0,00	1,00	1,00	0,04	0,00	0,00	1,00	0,00
C	0,00	0,20	1,00	0,01	1,00	0,01	1,00	0,03	0,02	1,00	0,00	1,00	0,01	0,50	0,04	0,04	1,00	0,00	0,85	0,01	0,08
D	0,00	0,00	0,01	1,00	0,08	0,00	0,14	0,00	0,00	0,00	1,00	0,11	0,00	1,00	0,00	0,00	0,00	1,00	1,00	0,00	1,00
E	0,00	0,00	1,00	0,08	1,00	0,00	1,00	0,00	0,00	1,00	0,11	1,00	0,00	0,01	0,00	0,00	0,85	1,00	1,00	0,00	0,06
F	1,00	1,00	0,01	0,00	0,00	1,00	0,00	1,00	1,00	0,01	0,00	0,00	1,00	0,00	1,00	1,00	0,01	0,00	0,00	1,00	0,00
G	0,00	0,00	1,00	0,14	1,00	0,00	1,00	0,00	0,00	0,50	1,00	0,01	0,00	1,00	0,00	0,00	0,08	1,00	0,06	0,00	1,00

Table S7 Occurrence results after Bonferroni correction for paired CLASS comparisons at each stage (p<0.05 highlighted)

STAGE	Training							PracticeA							PracticeB						
Class comparison	A	B	C	D	E	F	G	A	B	C	D	E	F	G	A	B	C	D	E	F	G
A	1,00	1,00	0,01	0,26	0,00	0,01	0,00	1,00	1,00	0,01	0,46	0,00	0,00	0,00	1,00	1,00	0,00	0,02	0,00	0,00	0,00
B	1,00	1,00	0,24	1,00	0,00	0,00	0,00	1,00	1,00	0,02	1,00	0,00	0,00	0,00	1,00	1,00	0,02	0,30	0,00	0,00	0,00
C	0,01	0,24	1,00	1,00	0,00	1,00	0,00	0,01	0,02	1,00	1,00	0,00	1,00	0,00	0,00	0,02	1,00	1,00	0,00	1,00	0,00
D	0,26	1,00	1,00	1,00	0,00	1,00	0,00	0,46	1,00	1,00	1,00	0,00	1,00	0,00	0,02	0,30	1,00	1,00	0,00	1,00	0,00
E	0,00	0,00	0,00	0,00	1,00	0,00	1,00	0,00	0,00	0,00	0,00	1,00	0,00	1,00	0,00	0,00	0,00	0,00	1,00	0,00	1,00
F	0,01	0,00	1,00	1,00	0,00	1,00	0,00	0,00	0,00	1,00	1,00	0,00	1,00	0,00	0,00	0,00	1,00	1,00	0,00	1,00	0,00
G	0,00	0,00	0,00	0,00	1,00	0,00	1,00	0,00	0,00	0,00	0,00	1,00	0,00	1,00	0,00	0,00	0,00	0,00	1,00	0,00	1,00

Table S8 Results of paired STAGE comparisons with Bonferroni correction at each microstate class for three types of microstate parameters

	STAGE comparisons	A	B	C	D	E	F	G
Coverage	Training-PracticeA	1,00	0,94	1,00	1,00	1,00	0,16	0,67
	Training-PracticeB	0,58	0,72	0,71	1,00	0,61	0,31	0,09
	PracticeA -PracticeB	1,00	1,00	1,00	0,63	0,51	1,00	1,00
Duration	Training-PracticeA	0,96	0,39	1,00	1,00	1,00	0,42	0,91
	Training-PracticeB	0,34	0,98	0,86	1,00	0,48	0,90	0,22
	PracticeA -PracticeB	0,54	1,00	1,00	1,00	0,21	1,00	0,96
Occurrence	Training-PracticeA	1,00	1,00	1,00	1,00	1,00	0,30	0,52
	Training-PracticeB	1,00	0,81	1,00	1,00	1,00	0,55	0,40
	PracticeA -PracticeB	1,00	0,64	1,00	0,29	1,00	1,00	1,00
